# Impact of unequal distances among acoustic sensors on cross-correlation based fisheries stock assessment technique

**DOI:** 10.1038/s41598-020-73079-z

**Published:** 2020-09-28

**Authors:** Shaik Asif Hossain, Monir Hossen

**Affiliations:** Department of Electronics and Communication Engineering, Khulna University of Engineering & Technology, Khulna, 9203 Bangladesh

**Keywords:** Computational biology and bioinformatics, Statistical methods

## Abstract

Cross-correlation based fisheries stock assessment technique utilized array of multiple acoustic sensors which were equidistant pair. However, at practical implementation of this technique, equal distances among acoustic sensors is sometimes challenging due to different practical phenomenon. Therefore, in this study, we work on this issue and investigated the impact of unequal distances among the acoustic sensors. We found that cross-correlation based technique proved its effectiveness even for the unequal spacing among acoustic sensors. We considered chirp generating species of fish and mammals, i.e., damselfish (*Dascyllus aruanus*), humpback whales (*Megaptera novaeangliae*), dugongs (*Dugong dugong*), etc., species, and three acoustic sensors array for simulation purposes. Some limitations including negligence of multipath interference, assuming the delays to be integer were compromised during simulations.

## Introduction

In worldwide, over 800 species of fish and mammals from 109 families are identified to be soniferous^1^, although this is likely to be an underestimate. Over 150 species of these are found in the northwest Atlantic^2^. Amongst the soniferous fish and mammals, some of the most abundant and vital commercial species are codfish, drum fish, grunts, groupers, snappers, jacks, catfish^3^, hambuck wheals, dolphins, etc.

Utilizing the sounds of fish and mammals is recognized as passive acoustics which has been used for over 50 years in fish biology and fisheries surveys^2^. In fact, it is used routinely to delineate and monitor spawning areas, and study the behavior of fish and mammals^4,5^. The researchers use hydrophone to listen this sound from fish and mammals to identify species^6,7^ and estimate their population size^5^. They use signal processing and spectral analysis computer algorithms to perform their tasks for this purpose^5,6^. Generally, passive acoustic monitoring is a technique which is distinguished from other types of bioacoustics since it utilizes naturally occurring sounds to gather information on fish and mammals, rather than using artificially generated sounds. It offers a number of benefits to survey fishery population in a certain marine area including being a method of non-optically observing fish activity and distribution, being a non-invasive and non-destructive observational tool, providing the capability of continuous or long-term monitoring as well as remote monitoring, etc^6^. In addition, investigators use passive acoustics to monitor the sources of noise pollution, and to study the impact of man's activities on marine communities^6^.

A straightforward, cross-correlation based population estimation technique was proposed in Refs.^8,9^, was a passive acoustic monitoring technique, can solve some major drawbacks of conventional fish population estimation techniques. Researchers investigated different practical impacts which were associated with this technique. Hossain and Hossen have investigated the impact of increasing number of acoustic sensors, i.e., increasing number of cross-correlation function (CCF) in Ref.^10^. Hossain and Hossen also have shown the effect of underwater bandwidth and SNR on this technique in Ref.^11^. Impact of dispersion coefficient on this technique was investigated in Ref.^12^. Similarly, impact of different distribution on estimation was demonstrated in Ref.^13^. Hossain and Hossen have investigated the procedure of calculating statistical error of this technique illustrated in Ref.^14^. However, in the former researches, all the researchers have considered equal distance among the acoustic sensors. This is sometimes impossible in some practical cases because it is somewhat troublesome to put the sensors at the desired locations in the randomly distributed fish and mammals. So, the constraint of equal spacing among sensors makes this technique difficult to implement practically, which is recognized as a limitation of this technique. With an aim to overcome this limitation, we started our investigation. In this paper, we have considered three acoustic sensors, where the sensors maintain unequal distances. Three acoustic sensors can be organized by two types of topologies, i.e., acoustic sensors in line (ASL) scheme and acoustic sensors in a triangle (AST) scheme^9^. We have worked with both schemes to investigate the impact of unequal distances among the acoustic sensors. From diverse types of fish sounds, we have considered chirp sound and use its frequency for simulations. This type of sound is very common in damselfish (*Dascyllus aruanus*), humpback whales (*Megaptera novaeangliae*), dugongs (*Dugong dugon*), etc., species. Firstly, we have worked to establish a theoretical impact and then we have verified the theory by simulation. In this research, MATLAB R2010a was used as our simulation tool.

## Background

In this section, the methodology of cross-correlation based population estimation technique^9,10,15^ is deliberated. We have considered three acoustic sensors array in this study. This type of array can be either ASL form or AST form as in Ref.^10^. In this section, first, we will describe the methodology of ASL scheme and then AST scheme. As we have considered chirp sound generating fish and mammals in this study, this section will describe the estimation process with respect to this chirp signal.

Let us consider a three-dimensional spherical area containing *N* fish and mammals and three acoustic sensors. In this area, the fish and mammals are evenly distributed over the whole region. The fish and mammals are the sources of chirp signals and the acoustic sensors are the receivers.

**Figure 1:**
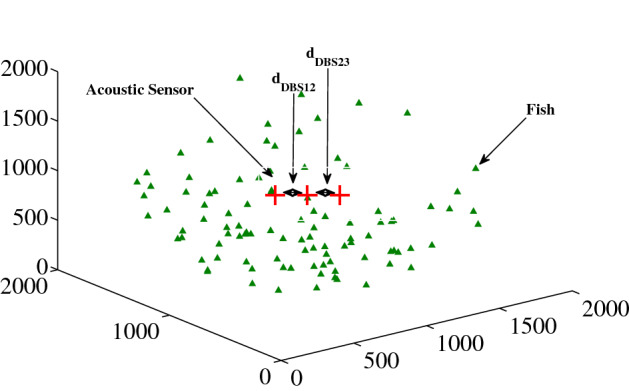
A uniform random distribution of fish and mammals with three equally spaced acoustic sensors using ASL scheme. Here, the axes units are in meter. We used MATLAB R2010a to achieve this figure.

In the ASL scheme, the three equally separated acoustic sensors (*H*_1_, *H*_2_ and *H*_3_) stay in a straight line at the center of the sphere as shown in Fig. 1. The distances among the acoustic sensors are such that, *d*_DBS12_ (distance between *H*_1_ and *H*_2_) = *d*_DBS23_ (distance between *H*_2_ and *H*_3_) = *d*_DBS_ (distance between the equidistant pair of acoustic sensors).

Now, if chirp signal is produced by a fish or mammal, it will be recorded by three sensors with the corresponding time delays and attenuations. Henceforth, we can express the CCFs of the chirp signals received at each pair of sensors individually by a delta function^16,17^. For each fish or mammal, we will find a delta function after cross-correlation. These delta functions inhabit any position in the sample space of the corresponding pair of acoustic sensors. Now, we can consider each delta function as a ball and the samples between the corresponding pair of acoustic sensors as the bins into which the balls may fall^16,17^.

**Figure 2:**
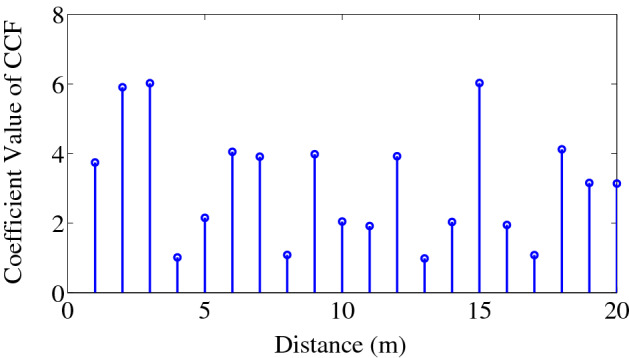
Bins, *b* in the cross-correlation function. We used MATLAB R2010a to achieve this figure.

We will define the number of bins, *b* (as shown in Fig. 2) as twice the number of samples between the acoustic sensors, minus one as^16,17^:

1 $$b = \frac{{2 \times d_{DBS} \times S_{R} }}{{S_{P} }} - 1.$$

Here, *S*_R_ is the sampling rate and *S*_P_ is the speed of propagation.

*R* of CCF is defined as the ratio of standard deviation (*σ*) to the mean (*µ*) is chosen as the estimation parameter of this process, as it requires no prior knowledge of the signal strength^16^. Two estimation parameters, *R*_12_ and *R*_23_ are derived from two CCFs, *C*_12_(τ) and *C*_23_(τ), respectively (where, τ is the time delay), to calculate the final estimation parameter of ASL scheme^10^. Then, the final estimation parameter, $R_{Equal:ASL}^{2CCF}$ of ASL scheme is obtained by taking the average of *R*_12_ and *R*_23_, and can be expressed as^10^:

2 $$R_{Equal:ASL}^{2CCF} = \frac{{R_{12} + R_{23} }}{2} = \frac{{\frac{{\sigma_{12} }}{{\mu_{12} }} + \frac{{\sigma_{23} }}{{\mu_{23} }}}}{2},$$

where, *σ*_12_ and *σ*_23_ represent standard deviation and *µ*_12_ and *µ*_23_ represent the mean of the CCFs, i.e., *C*_12_(τ) and *C*_23_(τ), respectively.

It is very complex to calculate the *R* of CCF using mathematical expressions. So, the cross- correlation related problem is reframed into a probability problem using the well-known occupancy problem^16^. After reframing, *R* of CCF can be written as^16^:

3 $$R = \frac{\sigma }{\mu } = \frac{{\sqrt {N \times \frac{1}{b} \times (1 - \frac{1}{b})} }}{\frac{N}{b}} = \sqrt {\frac{b - 1}{N}} .$$

Hence, Eq. (2) can be expressed as^10^:

4 $$R_{Equal:ASL}^{2CCF} = \frac{{R_{12} + R_{23} }}{2} = \frac{{\sqrt {\frac{{b_{12} - 1}}{N}} + \sqrt {\frac{{b_{23} - 1}}{N}} }}{2}.$$

Here, *b*_12_ and *b*_23_ denote the number of bins of the CCFs, *C*_12_(τ) and *C*_23_(τ), respectively. In this case, *b*_12_ = *b*_23_ = *b* according to Eq. (1); as the values of *S*_R_ and *S*_P_ are constant during the estimation process and *d*_DBS12_ = *d*_DBS23_ = *d*_DBS_. Hence, Eq. (4) becomes

5 $$R_{Equal:ASL}^{2CCF} = \frac{{R_{12} + R_{23} }}{2} = \sqrt {\frac{b - 1}{N}} .$$

Using Eq. (5), we can estimate *N*, as *b* is known from Eq. (1) and $R_{Equal:ASL}^{2CCF}$ can be calculated from the CCFs.

**Figure 3:**
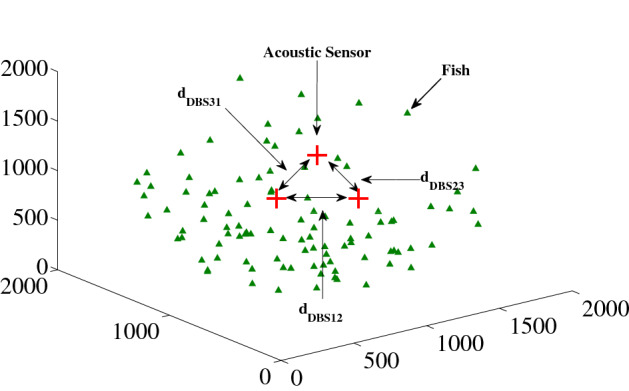
A distribution of fish and mammals with three equally spaced acoustic sensors for AST scheme. Here, the axes units are in meter. We used MATLAB R2010a to achieve this figure.

In AST scheme, the three sensors (*H*_1_, *H*_2_ and *H*_3_) form an equilateral triangle for estimation purpose, where the centroid of that triangle stays at the center of the sphere as shown in Fig. 3. So, the distances among the acoustic sensors are such that, *d*_DBS12_ (between *H*_1_ and *H*_2_) = *d*_DBS23_ (between *H*_2_ and *H*_3_) = *d*_DBS31_ (between *H*_3_ and *H*_1_) = *d*_DBS_ (between the equidistant pair of acoustic sensors).

In the AST scheme, three CCFs, *C*_12_(τ), *C*_23_(τ), and *C*_31_(τ) are used for estimation^10^. The estimation parameters, *R*_12_, *R*_23_, and *R*_31_ are derived from three CCFs, i.e., *C*_12_(τ), *C*_23_(τ), and *C*_31_(τ), respectively, to calculate the ultimate estimation parameter of the AST scheme. Then, the estimation parameter $R_{Equal:AST}^{3CCF}$ of the AST scheme is obtained by averaging the *R*_12_, *R*_23_, and *R*_31_, and can be expressed as^10^:

6 $$R_{Equal:AST}^{3CCF} = \frac{{R_{12} + R_{23} + R_{31} }}{3} = \frac{{\frac{{\sigma_{12} }}{{\mu_{12} }} + \frac{{\sigma_{23} }}{{\mu_{23} }} + \frac{{\sigma_{31} }}{{\mu_{31} }}}}{3},$$

where, *σ*_12_, *σ*_23_, and *σ*_31_ signify the standard deviation and *µ*_12,_ *µ*_23_, and *µ*_31_ signify the mean of the CCFs, i.e., *C*_12_(τ), *C*_23_(τ), and *C*_31_(τ), respectively.

Similar to Eq. (4), we can write

7 $$R_{Equal:AST}^{3CCF} = \frac{{R_{12} + R_{23} + R_{31} }}{3} = \frac{{\sqrt {\frac{{b_{12} - 1}}{N}} + \sqrt {\frac{{b_{23} - 1}}{N}} + \sqrt {\frac{{b_{31} - 1}}{N}} }}{3}.$$

Here, *b*_12,_ *b*_23_, and *b*_31_ represent the number of bins of the CCFs, i.e., *C*_12_(τ), *C*_23_(τ), and *C*_31_(τ), respectively. Since the acoustic sensors are equidistant pair and *b*_12_ = *b*_23_ = *b*_31_, we can rewrite the Eq. (7) as^10^

8 $$R_{Equal:AST}^{3CCF} = \frac{{R_{12} + R_{23} + R_{31} }}{3} = \sqrt {\frac{b - 1}{N}} .$$

## Impact of unequal distances among acoustic sensors

The previous section showed the estimation process with equal distances among the acoustic sensors. This section will investigate a theoretical and simulated impact of unequal distance among the acoustic sensors on population estimation. At first, we will investigate the theoretical approach and then simulated approach to justify the theory.

Here, from diverse types of fish sounds, we have considered chirp sound which is usually generated by damselfish (*Dascyllus aruanus*)^18^, humpback whales (*Megaptera novaeangliae*)^19^, dugongs (*Dugong dugon*)^20^, etc., species. A sound analysis of *Plectroglyphidodon lacrymatus* and *Dascyllus aruanus* species of damselfish (family pomacentridae) demonstrated that their generated chirp sounds consisted of trains of 12–42 short pulses of three to six cycles, with a durations from 0·6 to 1·27 ms; and the peak frequency varied from 3400 to 4100 Hz illustrated in Ref.^21^. The expression of this signal is found in Refs.^9,10,15^ as:

9 $$X(t) = A\cos \left[ {\left\{ {2\pi \left( {\frac{{(f_{2} - f_{1} )t^{2} }}{2d} + f_{1} t} \right)} \right\} + P} \right],$$

where, *f*_1_ is the starting frequency in Hz, *f*_2_ is the ending frequency in Hz, *d* is the duration in second, *P* is the starting phase, and *A* is the amplitude.

**Figure 4:**
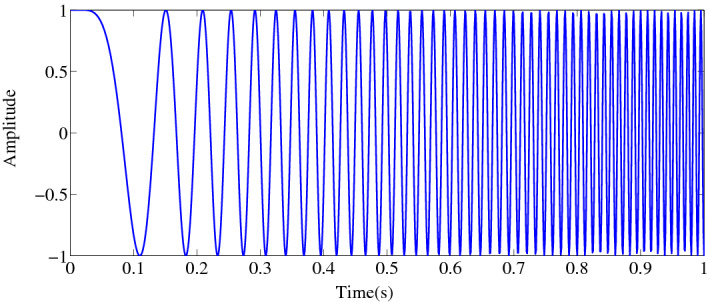
Chirp signal from simulation. We used MATLAB R2010a to achieve this figure.

Figure 4 shows simulated form of chirp signal which represents a simple form of chirp with duration of 1 s.

### Theoretical impact

In this subsection, we will describe the theoretical impact of unequal distances among the acoustic sensors on cross-correlation based population estimation technique. Firstly, we will investigate the theory for the ASL and AST schemes.

Three unequally spaced sensors denoted by *H*_1_, *H*_2_, and *H*_3_ are considered along a line for estimation. Here, the middle sensor *H*_2_ is placed at the center of the estimation area. Unequal distances signify that *d*_DBS12_ ≠ *d*_DBS23_ ≠ *d*_DBS31_. However, the process is analogous to the estimation process described in “Background”.

Therefore, we can write

10 $$R_{Unequal:ASL}^{2CCF} = \frac{{R_{12} + R_{23} }}{2} = \frac{{\sqrt {\frac{{b_{12} - 1}}{N}} + \sqrt {\frac{{b_{23} - 1}}{N}} }}{2}.$$

Now, *b*_12_ and *b*_23_ can be expressed using Eq. (1) as:

11 $$b_{12} = \frac{{2 \times d_{DBS12} \times S_{R} }}{{S_{P} }} - 1.$$

And,

12 $$b_{23} = \frac{{2 \times d_{DBS23} \times S_{R} }}{{S_{P} }} - 1.$$

It is not possible to simplify Eq. (10) because *b*_12_ ≠ *b*_23_ and *S*_R_ and *S*_P_ are fixed. Hence, Eq. (10) will be

13 $$N = \left( {\frac{{\sqrt {b_{12} - 1} + \sqrt {b_{23} - 1} }}{{2 \times R_{Unequal:ASL}^{2CCF} }}} \right)^{2} .$$

Now, to establish a relationship between equal and unequal distances among the acoustic sensors, if we consider the ratio $\frac{{R_{Equal:ASL}^{2CCF} }}{{R_{Unequal:ASL}^{2CCF} }}$, i.e.,$\frac{{\sqrt {\frac{b - 1}{N}} }}{{\frac{{\sqrt {\frac{{b_{12} - 1}}{N}} + \sqrt {\frac{{b_{23} - 1}}{N}} }}{2}}}$, equals 1, we find

14 $$\frac{{2 \times \sqrt {b - 1} }}{{\sqrt {b_{12} - 1} + \sqrt {b_{23} - 1} }} = 1.$$

After reframing Eq. (14), we can find

15 $$b = \frac{{(\sqrt {b_{12} - 1} + \sqrt {b_{23} - 1} )^{2} + 4}}{4}.$$

This is the condition between the equal and unequal sensor separation cases among the bins for three acoustic sensors in the ASL scheme.

However, there is another way to establish a special condition for this scheme. If we consider *C*_31_, i.e., CCF due to cross-correlation between acoustic signals received at *H*_3_ and *H*_1_, another condition will be found. This CCF is not considered in equal sensor separation case, as only the CCFs due to the equidistant pair of sensors are used for that case. Considering the additional CCF, *C*_31_(τ), the ultimate estimation parameter of the ASL scheme with unequally separated sensors can be achieved by averaging the estimation parameters, *R*_12_, *R*_23_, and *R*_31_, derived from the CCFs, *C*_12_(τ), *C*_23_(τ), and *C*_31_(τ), respectively, can be expressed as:

16 $$R_{Unequal:ASL}^{3CCF} = \frac{{R_{12} + R_{23} + R_{31} }}{3} = \frac{{\sqrt {\frac{{b_{12} - 1}}{N}} + \sqrt {\frac{{b_{23} - 1}}{N}} + \sqrt {\frac{{b_{31} - 1}}{N}} }}{3}.$$

Here, *b*_31_ is the number of bins of *C*_31_(τ), which can be written as

17 $$b_{31} = \frac{{2 \times d_{DBS31} \times S_{R} }}{{S_{P} }} - 1.$$

In the ASL scheme, *d*_DBS31_ = *d*_DBS12_ + *d*_DBS23_ and *b*_12_ ≠ *b*_23_ ≠ *b*_31_. Hence, from Eq. (17), we can write as

18 $$b_{31} = b_{12} + b_{23} + 1.$$

Therefore, from Eq. (16), we can write

19 $$R_{Unequal:ASL}^{3CCF} = \frac{{\sqrt {\frac{{b_{12} - 1}}{N}} + \sqrt {\frac{{b_{23} - 1}}{N}} + \sqrt {\frac{{b_{12} + b_{23} }}{N}} }}{3}.$$

After reframing Eq. (19), we can write

20 $$b = \frac{{(\sqrt {b_{12} - 1} + \sqrt {b_{23} - 1} + \sqrt {b_{12} + b_{23} } )^{2} + 9}}{9},$$

which implies a special condition for unequal distances among acoustic sensors using three sensors in ASL scheme.

Now, we will establish a condition for unequal distances among acoustic sensors using three sensors in AST scheme. Analogous to special condition of ASL, AST scheme also has the similar property, i.e., *d*_DBS31_ = *d*_DBS12_ + *d*_DBS23_ and *b*_12_ ≠ *b*_23_ ≠ *b*_31_.

If we take the ratio $\frac{{R_{Equal:AST}^{3CCF} }}{{R_{Unequal:AST}^{3CCF} }}$ equals 1, we will find

21 $$\frac{{\sqrt {\frac{b - 1}{N}} }}{{\frac{{\sqrt {\frac{{b_{12} - 1}}{N}} + \sqrt {\frac{{b_{23} - 1}}{N}} + \sqrt {\frac{{b_{31} - 1}}{N}} }}{3}}} = \frac{{3 \times \sqrt {b - 1} }}{{\sqrt {b_{12} - 1} + \sqrt {b_{23} - 1} + \sqrt {b_{31} - 1} }} = 1.$$

After reframing Eq. (21), we can write

22 $$b = \frac{{(\sqrt {b_{12} - 1} + \sqrt {b_{23} - 1} + \sqrt {b_{31} - 1} )^{2} + 9}}{9}.$$

This is the condition for the equal and unequal sensor separation cases among the bins for three acoustic sensors of the AST scheme.

### Simulated impact

In this subsection, we will verify the theory by using simulated results. A MATLAB simulation environment was considered to obtain the simulations of proposed scheme. To acquire simulations, a uniform random distribution of fish and mammals was considered. The entire simulations were obtained with respect to chirp generating fish and mammals. No signal parameters were changed in the simulation. We have generated fish sounds considering different real-time parameters, i.e., frequency, time duration, bandwidth, etc.^9^. Similarly, fish signals can be represented as swift frequency wave^9^. The toolbox of MATLAB provides functions to generate swept-frequency waveforms such as the chirp function.

The following parameters were used in the MATLAB simulation as stated in Table 1. The motivation of this parameter setting is to give a clear idea about the total simulation, i.e., procedure and results, of this research.

**Table 1:** Parameters used in the MATLAB simulation.

Parameters	Value
Dimension of the sphere	2000 m
Distance between the acoustic sensors	0.45 m, 0.675 m, 0.95 m, 1 m, 1.275 m, 1.3 m, 1.5 m, 2 m, 2.075 m (can be varied)
Speed of propagation, *S*_*P*_	1500 m/s
Sampling rate, *S*_*R*_	60 kSa/s
Absorption coefficient, *a*	1 dBm^−1^
dispersion factor, *k*	0
Number of bins, *b*	17, 26, 37, 39, 50, 51, 59, 79, 82 (can be varied)
Number of iterations	500
Considered distribution	Uniform random distribution

A negligible amount of power difference among the acoustic pulses transmitted by each fish or mammal was considered to ease the simulations. We have used 500 iterations to achieve these simulated results, which mean the simulations run for 500 times and then average to achieve the plots. A SNR of 30 (26.02059 dB SNR) was considered to accomplish the simulation^11^

**Figure 5:**
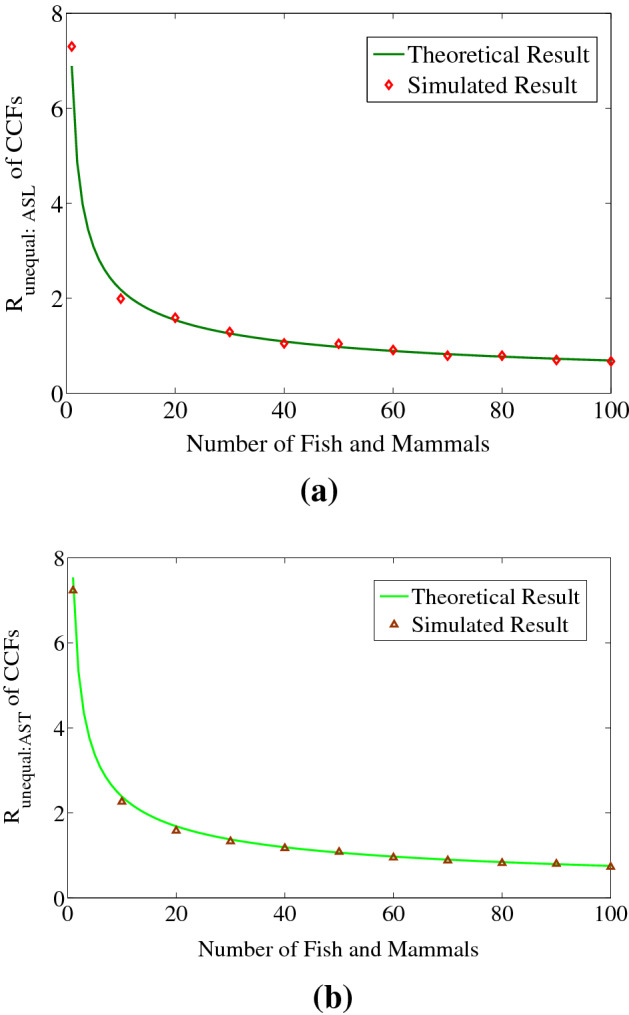
Number of fish and mammal’s vs. *R* of CCFs for unequal distances among acoustic sensors, (**a**) three acoustic sensors ASL scheme where, *d*_DBS12_ = 1 m and *d*_DBS23_ = 1.5 m and (**b**) three acoustic sensors AST scheme where, *d*_DBS12_ = 1 m, *d*_DBS23_ = 1.5 m and *d*_DBS31_ = 2 m. We used MATLAB R2010a to achieve these figures.

Figure 5 shows the comparison of simulated and theoretical results. Figure 5a corresponds to the ASL scheme of three acoustic sensors and Fig. 5b corresponds to the AST scheme of three acoustic sensors to show the effect of unequal sensor separations. The values of *d*_DBS12_ = 1 m and *d*_DBS23_ = 1.5 m; *b*_12_ = 39 and *b*_23_ = 59 in Fig. 5a. The values of *d*_DBS12_ = 1 m, *d*_DBS23_ = 1.5 m and *d*_DBS31_ = 2 m; *b*_12_ = 39, *b*_23_ = 59 and *b*_23_ = 79. It is seen from the Fig. 5 that the simulated results are very close to the theoretical results. This signifies the effectiveness of cross-correlation based passive monitoring method in the case of unequal distances among the acoustic sensors. Now simulations are performed to validate the conditions which we achieved in Eqs. (15) and (22). Analogous to the Fig. 5, simulated results of $R_{Unequal:AST}^{3CCF}$ are plotted against the number of fish and mammals.

**Figure 6:**
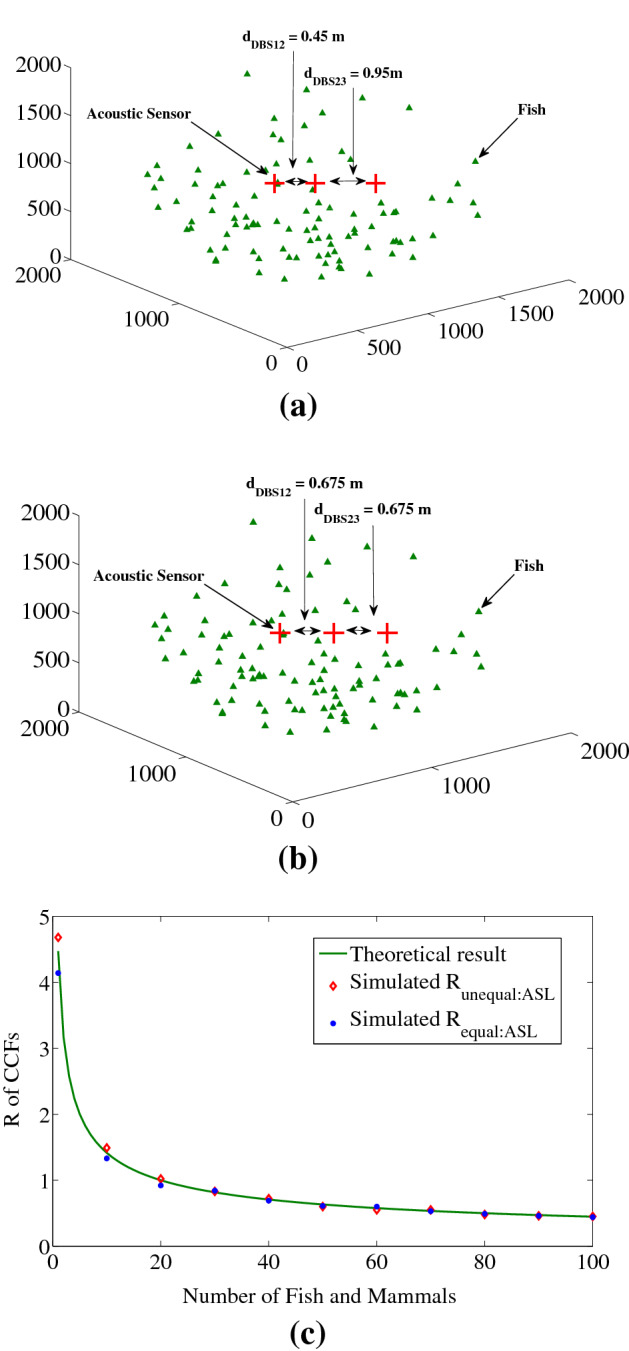
Scenario of unequal sensor separations and its corresponding results from simulations for three acoustic sensors ASL scheme (**a**) unequal sensor distances among acoustic sensors where *d*_DBS12_ = 0.45 m and *d*_DBS23_ = 0.95 m, (**b**) corresponding equal distances among acoustic sensors, i.e., *d*_DBS_ = 0.675 m, derived from condition at Eqs. (15) and (20) and (**c**) Number of fish and mammals vs. *R* of CCFs for theoretical, and simulated with unequally and corresponding equally spaced acoustic sensors where the sensor distances are similar as in (**a**) and (**b**). Here, the axes units are in meter for (**a**) and (**b**). We used MATLAB R2010a to achieve this figure.

The values used to find the results in Fig. 6c are similar as scenario shows in Fig. 6a,b, i.e., *d*_DBS12_ = 0.45 m (*b*_12_ = 17), *d*_DBS23_ = 0.95 m (*b*_23_ = 37) for unequal separation case and *d*_DBS_ = 0.675 m (*b* = 26) for corresponding equal separation case.

**Figure 7:**
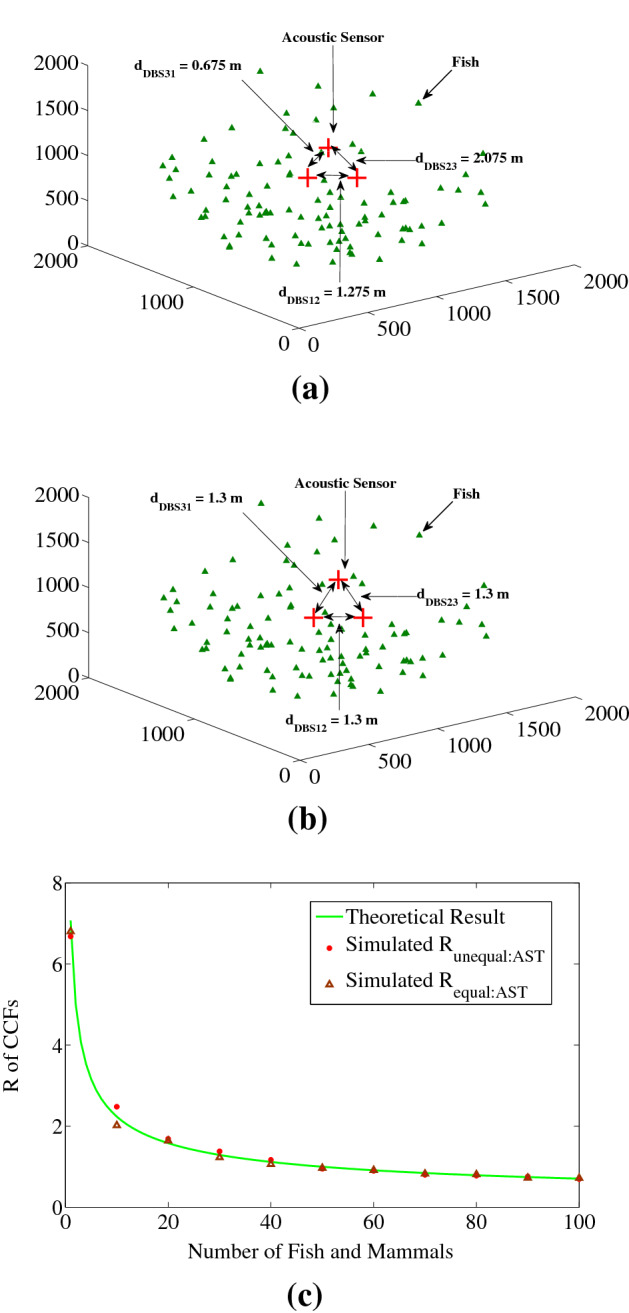
Scenario of unequal sensor separation and its corresponding results from simulations for three acoustic sensors AST scheme (**a**) unequal sensors separation where *d*_DBS12_ = 1.275 m, *d*_DBS23_ = 2.075 m and *d*_DBS31_ = 0.675 m (**b**) corresponding equal distance among acoustic sensors, i.e., *d*_DBS_ = 1.3 m, derived from condition at Eq. (22) and (**c**) Number of fish and mammals vs. *R* of CCFs for theoretical, and simulated with unequally and corresponding equally spaced acoustic sensors where the sensor distances are similar as in (**a**) and (**b**). Here, the axes units are in meter for (**a**) and (**b**). We used MATLAB R2010a to achieve these figures.

Figure 7 is simulated for the similar purpose as Fig. 6 but for three acoustic sensors AST scheme. Figure 7a shows an unequal sensor separation case of AST scheme where *d*_DBS12_ = 1.275 m, *d*_DBS23_ = 2.075 m and *d*_DBS31_ = 0.675 m whereas Fig. 7b shows corresponding equal sensor separation case of AST scheme with the distances among equidistant sensors is 1.3 m. Figure 7c shows the variation of simulated results of unequal sensor separation case from corresponding equal sensors separation case with respect to theoretical results. The values used to find Fig. 7c are similar as scenario shows in Fig. 7a,b, i.e., *d*_DBS12_ = 1.275 m (*b*_12_ = 50), *d*_DBS23_ = 2.075 m (*b*_23_ = 82) and *d*_DBS31_ = 0.675 m (*b*_31_ = 26) for unequal separation case and *d*_DBS_ = 1.3 m (*b* = 26) for corresponding equal separation case.

We can see from Figs. 5, 6, and 7, the simulated results closely agreed with the theoretical results. Therefore, these results demonstrate the robustness of estimation even with unequally sensors separation. At the same time, the effectiveness of the conditions derived in Eqs. (15) and (22) is proved.

However, this research has certain bounds, i.e., negligence of the effect of multipath interference, consideration of uniform random distributions, assumption of a negligible amount of power difference among acoustic pulses, assuming the delays to be integer, and consideration of acoustic sensors to be stayed at the middle of the estimation area. Similarly, as a passive acoustic method, this technique can be only applicable to the soniferous species.

During practical implementation of this technique, several matters should be taken in to account, i.e., dispersion factor, limited bandwidth problem, proper signal to noise ratio (SNR), etc. Dispersion coefficient is the main factor of the distance dependent attenuation It was found that with the increase of dispersion coefficient, i.e., dispersion loss in the medium, a significant amount of deviation occurred from actual fishery quantity to the estimated^12^. Limited bandwidth of the underwater channel poses a barrier during acquisition of fish signals, which has infinite bandwidth. To overcome this problem, a proper scaling is a mandatory task^11^. Similarly, a low signal to noise ratio (SNR) is also an impediment to obtain an accurate estimation. It was found that estimation with minimum 26.02 dB SNR can perform like the noiseless estimation^11^. Researches are underway to overcome the barrier of center placement of acoustic sensors.

Many things can affect the speed of sounds, e.g., nature of the medium, (gas, liquid or solid), temperature, additive substances, i.e., such as salt in water, etc. Sounds travel faster through denser and hotter materials. At normal temperature, sound speed is 1493 ms^−1^ in fresh water, which is 1533 ms^−1^ in sea water^22^. However, in our research, we consider the propagation speed of fish sound is 1500 ms^−1^ during simulations

## Conclusion

The researchers of cross-correlation based passive monitoring technique considered equidistant acoustic sensors during their investigations previously. This consideration was a limitation of this estimation process. In this research, we have worked to eliminate this limitation of equidistant sensors. We also have worked with three acoustic sensors and derived two conditions, i.e., one regular and one special condition, for ASL scheme and one condition for AST scheme. We found that the acoustic sensors can be unequally spaced but in a way that verifies these two conditions. The conditions for both ASL and AST schemes are verified by simulations. We also have proved the robustness of this estimation process in the case of unequal sensor separation. Finally, it was our key goal to remove the barrier of the assumption of equal distance among the acoustic sensors and we have removed that in this study which satisfies our goals properly.
